# Iranian women’s psychological responses to positive HPV test result: a qualitative study

**DOI:** 10.1186/s12905-021-01272-x

**Published:** 2021-03-26

**Authors:** Kowsar Qaderi, Seyedeh Tahereh Mirmolaei, Mehrnaz Geranmayeh, Shahrzad Sheikh Hasani, Farnaz Farnam

**Affiliations:** 1 Reproductive Health Department, School of Nursing and Midwifery, Tehran University of Medical Sciences, Eastern Nosrat St. Tohid Sq., 141973317 Tehran, Iran; 2 Gynecology Oncology Department, Imam Khomeini Hospital Complex (IKHC), Tehran University of Medical Sciences (TUMS), Tehran, Iran

**Keywords:** Cervical cancer, Co-testing, Cervical screening, Female, Human papillomavirus, HPV testing, Psychological, Sexual health, Sexually transmitted infections

## Abstract

**Background:**

Human papillomavirus testing as an established screenings test allow for the early detection and treatment of cervical cancer. Testing positive for HPV may have adverse consequences for women. This study aimed to explore the psychological impacts of testing positive for HPV on women in a developing country with a distinct cultural and religious background.

**Methods:**

Qualitative face-to-face semi-structured interviews were conducted with 40 Iranian women who received a positive high-risk HPV result. Content analysis approach was used to data analysis through MAXQDA10.

**Results:**

Three main categories were emerged: initial confrontation; STD-related psychological burden; and rebuilding health. Initial reactions to positive HPV results were shock, unrealistic fear, confusion, distress, and financial concerns. Stigma was manifested in form of self-blame, fear of HPV-disclosure, negative body image, being stigmatized by healthcare providers, and receiving health care anonymously. Refusal to use insurance services showed how evident and powerful the stigma was. Most women reported lifestyles and sexual behaviors modifications to help their immune system to clear HPV; indicating that the screening can work as a valuable opportunity to improve women's physical and sexual health. Regular follow-up, safe sex and a focus on spirituality enable women infected with HPV to take control of the situation. Worrying about other HPV-linked cancers (oropharynx and anal) and fears of partner infection indicated that women consider HPV to be more than just a cause of cervical cancer.

**Conclusions:**

The findings implied to the HPV-positive women's need to support and factual information. Designing and implementing interventions that mitigate the psychological effect of positive HPV test results can highlight the potential benefits of screening for women's health.

## Background

HPV is a globally common virus in the sexually active population that can lead to certain types of cancers, including cervix, vagina, and vulva in women, penis in men, and anus, and back of throat in both women and men [1]. The only type of HPV-related cancer with a recommended screening test is cervical cancer (CC), due to its cause-and-effect relationship with the high-risk HPV (hrHPV) types [2]. Cervical cancer screening programs have been modified in recent years to decrease the CC incidence ratio [3]. The latest revision (2019) of guidelines for the management of cervical cancer screening abnormalities, conducted by the fourth American Society of Colposcopy and Cervical Pathology (ASCCP)-sponsored consensus, was motivated by the intricacy of the 2012 guidelines (concurrent HPV and cytology testing, known as co-testing) and the queue of about-to-be-available new test [4]. Updated guidelines were needed to accommodate the present available cervical screening strategies, namely primary human papillomavirus screening, co-testing, and cervical cytology alone [5]. Existing evidence suggests that, although hrHPV testing reaches the maximum level of sensitivity, specificity, and accuracy in cervical screening [3], it may result in significant psychological consequences due to the sexually transmitted nature of the virus [6–11].

There are several limitations in the available literature. To date, although extensive qualitative research has explored women's adverse psychological responses to HPV positive results, most of studies were conducted in the countries where HPV is common and HPV vaccination programmes have been introduced [12].

Despite the availability of the vaccine, national HPV vaccination programmes are very rare in the Middle East: only 1 country (the United Arab Emirates) has effectively introduced the vaccine through a national programme and very few others have intended to implement it in the near future [13]. In Iran, an HPV vaccination programme has not been launched, but Gardasil can be purchased in some pharmacies for about $ 100. The region of the Middle East and North Africa has historically been more shielded from HPV infections due to more conservative sexual behavior in this region. However, an unexpected rise in HPV infections in women has recently occurred due to an increase in individuals aged 10–24 years engaging in premarital sexual activity [14]. It is expected that the number of invasive cervical cancer cases will drop significantly with the implementation of consistent vaccinations, and that the most invasive cervical cancer cases will come from low- and middle-income countries by 2050 [14].

In Iran, according to Globocan (2020), age-standardized incidence rate for cervical cancer and mortality is 2.3 and 1.5 in 100,000 individuals, respectively [15]. It has been reported that cervical cancer screening participation among Iranian women aged 30 through 49 years is 53% (self-reported lifetime prevalence) [16]. The last cervical cancer screening recommendation in Iran is co-testing strategy for women aged 30–59, every five years [17]. Co-testing is available in all provinces of Iran although it is not covered by government insurance. So, most women have screening but pay for it by private insurance. There is a lack of comprehensive and reliable statistics about the proportion of women with a positive test for a hrHPV that go on to be diagnosed with pre-invasive or invasive cervical disease in Iran. Although a number of studies have reported on women’s views and experiences following a positive HPV test, few are from countries with the relatively low HPV positivity rates (approx. 7.4% as reported by the authors in Iran) [18], nor from countries with the specific cultural and religious contexts that will likely shape women’s reactions and responses [19]. In Iran, sex is culturally and religiously acceptable only to those whose marriages are legally registered. Even though, in large cities, adherence to these norms may be less, testing for an STI can be challenging for most Iranian women. Therefore, we conducted interviews with Iranian women who had undergone co-testing and received hrHPV-positive results to identify women’s psychological responses to HPV positivity.

## Methods

A qualitative study was conducted based on the conventional content analysis approach to understand the psychological responses of women to positive result of HPV test [20, 21].

This study was carried out at a referral gynecology-oncology outpatient clinic (equipped with colposcopy and directed by oncologist-gynecologist SSH and her colleagues), which serves a large population of women from all around the country with diverse backgrounds, and socio-economic status. The services in this clinic (including colposcopy) are very low-priced and offered to all patients, regardless of their insurance coverage.) The clinic is located in a large, busy, university-based, and geographically accessible complex, at the center of Tehran, the capital of Iran.

There is no unified HPV-related educational content (or leaflet) for Iranian women undergoing HPV testing. Health care providers who perform sampling or interpret the result for women will explain HPV to them. As a result, what women learn from their provider about HPV may vary in quality and quantity in different settings. All women referred to clinic with their Pap smear and hrHPV-positive results (following CCSP^1^) were sent by a coordinator of the clinic to the interviewer (KQ-Female-None of the participants were from the interviewer's region and there was no relationship between her and interviewees). KQ provided women with information about the purposes and methods of the study in a specific private, convenient room. She then obtained their informed consent. Women were eligible for interview if they received hrHPV positive results (either only hrHPV or both high-risk/low-risk strains) in the previous 6 months; were over 18 years of age with a heterosexual partnership; had no severe disease and were willing to share their experiences. Pregnant women were excluded. The non-random purposeful sampling was used to ensure heterogeneity with respect to age, marital status, education, and socioeconomic status. In total, 40 Persian-speaking women with diverse ethnic, cultural, and religious backgrounds were included. Their HPV typing and cytology results are shown in Table 1. None of them had cervical cancer at the time of their interview. Only two eligible women refused to participate because they prefer not to talk about HPV. Since the clinic is crowded, all participants interviewed during their waiting hours. One-to-one interviews were conducted using an interview guide (see Additional file 1: Appendix) which began with demographic and reproductive background and screening history. Three pilot-interviews were conducted (included in the study) to improve questions. Memos helped design the next questions to be asked from the following participants. Besides, field notes were made during the interviews. Interviews lasted between 35 and 90 min and were recorded with the consent of the participants. At the end of each interview, the audio file was playing two-to-three times (using hands-free), transcribed verbatim, and once again the audio file was listened to again to check the accuracy of the text. The data analysis was performed concurrently with data collection, using a qualitative content analysis approach with the aid of a trial version of MAXQDA 10 software. Initially, interview transcripts, field notes, and memos were integrated, and two coders (KQ and STM) read them several times to formulate a general understanding of the whole data. Based on this approach, open coding was conducted. One thousand two hundred twelve codes were then reduced by constant comparison and combination with each other. While the similar meaning codes were brought together into subcategories, the subcategories were compared and contrasted, and those with similar content were interpreted in a higher level of abstraction into the main categories. Therefore, the data-driven categories gradually emerged inductively and directly from the textual data. Data saturation was achieved over fifteen months, from September 2018 to December 2019. Some interviews were conducted in two sessions.

**Table 1:** HPV genotypes and cytology results of Interviewees

HPV genotypes and pap smear results of interviewees (n = 40)					
HPV genotypes	Pap test results				N
	Normal	ASCUS^a^	LSIL^b^	HSIL^c^	
High risk	7 (17.5%)	6 (15%)	6 (15%)	2 (5%)	21 (52.5%)
Mixed (low and high risk)	6 (15%)	6 (15%)	5 (12.5%)	2 (5%)	19 (47.5%)
N	13 (32.5%)	12 (30%)	11 (27.5%)	4 (10%)	40 (100%)

^a^Atypical squamous cells of undetermined significance

^b^Low-grade squamous intraepithelial lesion

^c^High-grade squamous intraepithelial lesion

The value of qualitative research can be judged according to its trustworthiness. To ensure trustworthiness, we used Lincoln and Guba’s four criteria: credibility, confirmability, dependability, and transferability [22, 23]. We provide credibility of the study results by prolonged engagement and member checking, by which, the transcript and extracted codes from the interview were returned to each interviewee to approve their accuracy. Member checking is a major step in establishing trustworthiness in qualitative research [24]. Lincoln and Guba [25] believe that member checking creates a level of trust between the researcher and the participants that results in mutual understandings and shared values. Conformability and dependability of the results were also established by peer debriefing and external checking. Therefore, all transcripts, codes, and categories were reviewed and rechecked by two supervisors. This process finally ended with several discussions between the research team about areas of disagreement until reaching a final consensus. We also tried to consider the maximum variation during sampling to enhance the transferability of the results. We interviewed women with diversity in age, relationship status, education, socioeconomic status, and cultural background. To ensure dependability, the process within the study was described in detail.

This study is coming from a PhD thesis in Reproductive Health titled: “HPV-positive women's concerns and needs: Development and evaluation of an intervention”, which was reviewed and approved by the Ethics Committee of School of Nursing related to Tehran University of Medical Sciences (IR.TUMS.FNM.REC.1397.139). Moreover, the authorities of hospital agreed to cooperate in the study. Direct quotes that are representative of the participants have been presented. The data (audio files) will be destroyed 18 months from the ending of this qualitative study (June 1st). All methods were carried out in accordance with relevant guidelines and regulations.

## Results

Participants' characteristics shown in Table 2 demonstrate the heterogeneity of the sample. Women ranged in age from 19 to 51 years, with the mean of 34.07 years. Details in parentheses following quotes represent the participant’s identification number, age (in years), and HPV typing (Mixed = both high-risk and low-risk).

**Table 2:** Demographic characteristics of participants

Demographic characteristics (n = 40)	
*Age*	
< 20 years	1 (2.5%)
20–30 years	5 (12.5%)
30–39 years	26 (65%)
40–49 years	7 (17.5%)
≥ 50 years	1 (2.5%)
*Marital status*	
Single	10 (25%)
Married	17 (42.5%)
Divorced/separated	10 (25%)
Widowed	3 (7.5%)
*Education level*	
Elementary	4 (10%)
Intermediary	12 (30%)
University—Bachelor	11 (27.5%)
University—Master or higher	13 (32.5%)
*Occupation*	
Housewife	15 (37.5%)
Employed	25 (62.5%)
*Months from receiving positive HPV results*	
Less than a month	10 (25%)
1–3 months	16 (40%)
4–6 months	14 (35%)
*Ethnicity*	
Persians	23 (57.5%)
Azeris	6 (15%)
Gilaks/Mazanis	4 (10%)
Kurds	3 (7.5%)
Lurs	2 (5%)
Baluch	1 (2.5%)
Arabs	1 (2.5%)
*Religious background*	
Muslim (Shi'a)	29
Muslim (Sunni)	3
Religious minorities	1 (2.5%)
Prefer not to answer	7

The results are presented in terms of three main categories: initial confrontation, STD-related psychological burden, and rebuilding health. Examples of codes and sub-categories are shown in Table 3.

**Table 3:** Psychological responses to positive HPV test results (Examples of the steps in the analysis)

Examples of transcript	Codes	Subcategories	Categories
“When I got home after learning of my 1 results, I started browsing the Internet until morning. I kept reading and crying. I was worried, I thought maybe I have Chlamydia or other STDs that I even don't know them, just like this one.” (P.9, 40, hrHPV)	Shock, Unexpected result, Denial, "Why me?" reaction Phobia, Catastrophizing, Fear of death, Distress, Psychosomatic symptoms	Primary psychological reactions	1. Initial confrontation
	Information seeking; stressful and confusing Search to find the source of infection Being vigilant about other STIs	Trying to understand the status quo	
	Concerns over the cervical cancer Fear of other HPV-associated cancers	Worries about HPV-related cancers	
	Not covering colposcopy, freezing and other STIs check-ups by insurance High cost of HPV vaccine	Financial concerns related to HPV management	
"Even if the government can cover 100% of HPV costs, many people do not benefit from it for fear of having their names recorded in insurance records. I don't even write my real name on the sample I give to the courier for transfer to the lab. I always ask the doctor's secretary to give me the last appointment so that the office is not crowded and no one sees me." (P.19, 38, hrHPV)	Shame, guilt and self-blame Negative body image Fear of social rejection Receiving health care anonymously Annoying behavior of health care providers	Stigma and Fear of disclosure	2. STD-related psychological burden
	Separation, Blame, Conflict, Rejection, Accusation Acceptance (past), Trust (non-sexual), Forgiveness (support)	Changing in emotional relationship	
	Fear of self-transmission to other parts of the body Self-protection by avoiding sexual contacts Worries about infecting partner Concerns over infecting family members non-sexually	Fear of spreading the virus	
"God likes me so much that it's been detected. I spent a lot of money on charity. I think I was getting this virus as a warning to be careful. I started reading "the Thanksgiving Miracle" book. I'm talking to my immune system just like a baby in my belly. My immune system will be able to combat the virus. I'll get rid of it soon."(P.3, 27, Mixed)	Remedies from alternative medicine Healthier food, Vitamins intake Modifying lifestyle Following up health status	Improving physical health	3. Rebuilding health
	Changing attitude toward the sexual issue Increasing Condom use Limiting sexual partners	Practicing safer sex	
	seeking and providing support Wishful thinking, Thankfulness, Hope for Heal	Growing spirituality and emotional well-being	

### Initial confrontation

All the participants in the study were asked about the initial experience of HPV positivity. Initial confrontation reflects their feelings and thoughts firstly after receiving a positive HPV result, consist of four categories.

#### Primary psychological reactions

As evidenced by the various respondents, the typical reactions to results were shock, fear, and distress. A 45-year-old widow recently tested positive for HPV stated: “My daughter said: 'why you are so scared?' I said: 'We are subconsciously afraid of everything we don't know about. To me, HPV is like jinn; unknown and scary!” (P.6, 45, Mixed).

A subdivision of participants has used coping mechanisms, such as denial. They generally rationalized their results with the probability of human errors of laboratory technicians. Some women did not expect their positive results due to being monogamous and having rigid hygienic habits.

Distress had led to losing appetite and sleep problems in several women. Some reported feeling overwhelmed by irrational fears and obsessive concerns. Several interviewees reported a disrupting of their roles (as an employee or student) due to distress and sadness. A single woman mentioned that severe anxiety and fear of death caused her neurotic back pain which led her to job loss. Few women, who all have received positive HPV results within the last month, had catastrophizing psychological conditions such as thinking of death and suicide.

#### Trying to understand the status quo

Almost all participants reported that they had little or no knowledge of HPV on the day of the test. After receiving positive results, they had tried to find out more about HPV on the Internet. They described the Internet as a private accessible source of comprehensive knowledge, but confusing and stressful as well. Confusion is well described as: “I was frantically searching. My mind was like a busy intersection, and I felt directionless.” (P.18, 41, Mixed).

Women commonly mentioned feeling uncertainty and confusion about the source and the time of contracting the virus. In most cases, the patient did not know whom to dump her misdirected anger. Most participants held their last partner accountable for infection (partner-directed guilt and anger). Women with mixed-status types stated that the partner having genital warts first was almost considered the source of infection.

Only two married women stated they had been cheating on their husband and accepted being accountable for the contamination (self-blame and self-directed guilt).

Acquiring virus from non-sexual sources (no anger, no blame, no guilt), as a defense mechanism or myth, mentioned only by married women with a long-term relationship that their husband claims being life-long monogamous.

Few stated that others (for instance, their previous partners or previous partners of the partner) were accountable for infection (deflected guilt and anger).

Some women revealed being vigilant about other STIs after getting information on the Internet. They worried that they might be infected with other STIs too.

#### Worrying about HPV-related cancers

Some women assumed that a positive hrHPV result means they definitely develop cervical cancer in the future. Women with more hrHPV genotypes, including 16/18 and abnormal cytology (CIN^2^) expressed intense fear and distress.

In our study, the main concerns of women with normal cervical cytology were other HPV-related cancers (throat and anal cancer). There is no approved test to determine HPV in the mouth, throat, and anus. Women revealed concerns over where exactly the virus exists. Women reporting participation in oro-genital practices were anxious about HPV potentials for causing oropharynx cancers. For instance, one woman reported she had mixed her salivary glands up with oral cancer. In response to these concerns, few said some laboratories had taken oral samples for HPV testing without a doctor's request, which is unnecessary and costly. It seemed women did not know what specialist they should see for sore in the mouth or anal canal. Questions about anal cancer mentioned by a woman complained of severe anal itching: “I think my anus is infected with the virus too. Is there a test like Pap smear for anal cells?” (P.29, 35, hrHPV).

#### Financial concerns related to HPV management

The majority of women mentioned that HPV testing, performing further procedures such as freezing or colposcopy, and following tests for other STIs (HIV, hepatitis B and C, and herpes) were expensive. “In the first weeks, it cost me three to four million Toman. Except for HIV test, other tests and procedures were costly since they're not covered by insurance.” (P.19, 38, hrHPV).

### STD-related psychological burden

Much of the adverse emotional reactions related to the sexually transmitted nature of HPV were escalated by religious values, cultural norms, and social rules. This category was strongly linked to the context of the study.

#### Stigma and fear of disclosure

Responses surrounding stigma were identified in different forms. A few participants, mostly single women, had assumed acquiring the virus as a punishment or repaying for individual mistakes. Three single women mentioned intense self-blame and guilt exacerbated by the self-perceived risk of cancer. They described it a devastating combination of shame and fear: “Dying of genital cancer due to a sexual disease is horribly embarrassing.” (P.11, 33, hrHPV).

A form of stigma experienced in this context was related to receiving health care anonymously. However, there are governmental-funded colposcopy clinics in almost every province; some women interviewed pointed out that they prefer not to use governmental services.

I didn't want to use my private insurance for colposcopy so that my family wouldn't know I have HPV. I think this story holds for so many girls because when I was in the doctor's office, I saw patients anxiously calling outside and borrowing money for colposcopy. (P.16, 36, Mixed)

I was recently hired at a health center. I'm afraid they will fire me if they find out I have HPV. (P.17, 32, hrHPV)

The majority of women reported that healthcare providers treat them with respect. Few women felt being stigmatized by healthcare providers and perceived as promiscuous and likely infected with other STIs. One of them reported:

When I took my mother to the gynecologist for HPV screening, Doctor said: 'Hajieh,^3^ This test is pertinent to women with risky behaviors!' and I felt so sorry for myself. (P.1, 33, hrHPV)

Another woman stated that her husband had been rejected by his urologist who was supposed to examine his warts: “as soon as he find out I have genital warts, he said without even looking at us, 'Get out of my office. I am not examining this disease!' And we got distraught.” (P.35, 42, Mixed).

As interviews showed, three-quarters of participants had revealed their results to at least one person (their current sexual partner, family member, or peers). Experiencing rejection and isolation, several women had regretted communicating their test results with others. They explained how misinformation about passing the virus through casual contacts would cause a social denial.

HPV positivity, whether with or without genital warts, had dramatically changed women’s feelings about their body image. Some women described feeling “disgusting”, “contaminated”, and “sexually unattractive”. Others reported a sense of “ugly” or “leper”.

Given the growing attention women have about their genitals' appearance, they suffer more in the presence of warts.

Touching my warts makes me sick. I don't want my partner to even look at them. I feel inadequate. I feel losing a part of my womanhood. I believe all the beauty of a woman is in her lips, breasts, and genitals. If one of these three has a problem, a woman gets devastated. (P.18, 41, Mixed)

#### Changing in emotional relationship

Few women had concealed their HPV from their partner. Concealment had led to receiving less financial support and going for appointments alone. These women were more likely to feel overwhelmed and helpless.

The consequences of disclosing the results with a partner were different. Separation due to the HPV was common among singles because it was considered as a sign of infidelity and being unhealthy. One woman stated that her divorce was mostly because of HPV. “He was not only a disgusting sex partner but also a traitor. Divorcing him, I protected myself from further infections.” (P.30, 31, Mixed).

A newlywed woman expressed disappointment as: “It just felt unfair! I feel I don't deserve to get an STD! I am so unlucky that after years of virginity, my first sexual relationship ended up like this!” (P.2, 34, hrHPV).

Very few women said they had been unfaithful. Considering themselves as the source of the infection, women often hid their HPV from their husband. Only a woman whose husband had noticed her infidelity, treated her with contempt and rejection.

A divorcee woman stated that her ex-husband accused her of deceiving him by hiding her incurable contaminating disease and divorced her.

Some couples tried to accept HPV experiencing painful emotional challenges and conflict. Women described it as an emotional dilemma. The women were finally able to forgive their husbands who attributed the virus to their ex- partners. HPV-positive women with normal cervical cytology, whose husband was responsible for the infection and took good care of them, had a milder psychological response to HPV.

#### Fear of spreading the virus

Worries about auto-inoculation were raised by some women. They thought they might spread the virus from one region of their body to another, (generally from the genitals to the mouth). Few women reported obsessive hygienic behaviors such as not using toilet paper and avoiding touching their genitals for fear of topical spreading virus to nearby tissues. “My doctor said that if I touch my genitals and then my mouth, it's like I had oral sex, and I have to avoid it, so I wear disposable gloves in the bathroom.” (P.29, 35, hrHPV).

Some women had stopped having sex; Most of them for the protection of themselves (self-protection) and a small number for the protection of their partners (partner-protection). Although some women reported using condoms usually, there was also worry and confusion about its protective role, which caused some of them not to have sex at all. Abstaining from sex was associated with concurrent "abnormal" cytology and hrHPV. These women maintain the physical act of sex would deteriorate their condition. “Realizing that sex is a risk factor of cervical cancer, I was more afraid of sex. My health is more important than sex.” (P.31, 36, Mixed, CIN2).

Women with mixed HPV genotypes and perineum warts were worried about internalizing more virus load from genital skin warts to their cervix during penetrative sex even with condoms.

A subset of women appeared to be more affected by concerns about risks associated with anal or oral sex or even kissing. It sometimes resulted in abstention from these practices.

Women who were sure they had lately gotten the virus (recently losing virginity or newly married women) were more likely to stop having sex for a while to clear the virus up. A woman stated that her decision was influenced by what they have learned about the virus: “HPV goes away on its own within six months to a year” and “re‐infection not allowing the virus to be cleared.” (P.23, 25, Mixed).

Some patients considered non-sexual modes of HPV contagion. They were nervous about passing the virus to others through casual contacts, surfaces, towels, clothes, and common utensils (spoon, mug, etc.). Participants who were mother noted that they were more concerned about infecting their children than serious complications of the virus, such as CC. Excessive hand washing has been reported by several women to protect families from contracting the virus. “Look at all dry cracks in my hands. I use bleach every day. My fingers are chapped and wounded. I was even thinking about living apart from my family so that my children wouldn't be infected.” (P.6, 45, Mixed).

### Rebuilding health

Despite a positive HPV result being distressing news for many women, it also has the potential to improve health outcomes. Interviews demonstrated that women had more focus on their physical, sexual, and spiritual health after receiving positive HPV results.

#### Improving physical health

Although HPV has no cure and it goes away by the person's immune system, interviews verified that some women consider strengthening their immune system to be a treatment for HPV. To increase the likelihood of virus clearance, they adopt lifestyles changes. Almost all participants reported arbitrary use of vitamins, minerals, mushrooms, etc.

I will accept anything if I can stay safe from cancer. I have bought everything anyone recommended to the extent that I need a daily plan for taking those. (P.12, 34, hrHPV)

Almost all women interviewed stated they want to continue regular CC follow-ups but few women believed identifying hrHPV means that they will not get CC. These women were less upset and had milder negative psychological response to HPV positivity.

Most women received positive HPV results 3–6 months before the interview reported lifestyle modifications to boost their immune system such as regular exercise, smoking cessation, reducing drinking habits, vegetarian diet, fasting, sugar cessation, healthy tea drinking, and adjusting sleep routines. These women were more likely to repeat the HPV test earlier than scheduled (12 months later) to check the effectiveness of the assumed treatment.

The majority of participants and their partners had taken one dose of the HPV vaccine and planned to complete their vaccination.

#### Practicing safer sex

Some participants reported that after receiving their positive HPV results, they had adopted a positive attitude towards safe sex and practiced sexual behaviors like abstinence, limiting the number of sexual partners, using condoms, and avoiding oral and anal sex.

#### Emotional support and growing spirituality

Women revealed a strong need for talking and receiving emotional supports from their significant others. “I needed to talk it out instead of keeping it bottled up.” (P.22, 35, hrHPV).

Several women felt less stigma and gained emotional support from being in online groups with other patients. Most participants felt better after the interview because they could ask their questions and talk to somebody listening to them carefully.

A subset of participants expressed some positive reactions such as cherishing the results to prevent worse outcomes like cervical cancer. Regular check-ups after receiving positive HPV test can be viewed as a blessing in disguise.

It may sound odd, but knowing I have HPV gave me a new idea or angle on things. I made some positive changes in my life. I think HPV may very well keep my family and me healthier and prevent other potential health issues or other STDs like HIV down the road. I am glad that I'm being vigilant about controlling what I can… (P.9, 40, hrHPV)

Women mentioned their religious beliefs, as a source of support that had helped them manage their stress. They hoped for healing and were grateful.

Thank God, I know it. My friend got ovarian cancer, and within three months, she went through a total hysterectomy. I'll never get cervical cancer because doctors can handle it at the precancerous stage. It's a bit of good luck. (P.5, 30, Mixed)

Two single women who felt deeply guilty talked about repentance. One stated: “I always say: God forgive me! I feel very helpless and I ask God to give me another chance! I pray that it will go away.” (P.12, 34, hrHPV).

## Discussion

This study provides an insight into the psychosocial responses of testing positive for HPV. In line with other studies, a positive HPV result was associated with stigma, distress, unrealistic fear of cancer, and concerns over deteriorating of emotional relationships [6, 26–29]. Distress had led to losing appetite, insomnia, and disrupting social roles in several women.

Excessive fear of cancer was seen in women who besides HPV (type 16/18), also had abnormal cytological results. Concern about other HPV-related cancers (throat and anal cancers) and fear of partner infection show that women consider HPV more than cervical cancer agent.

Similar to another study [30], Internet was the primary source of information for Iranian women, due to the absence of a formal and integrated HPV-information programme. There is a need for review of the information materials provided to women with HPV. Additionally, it is recommended to launch a website in the native language containing up-to-date and useful content (translated from reliable websites like CDC^4^ and WHO^5^). So hHealth professionals can refer HPV patients and the general public to this site. The content of this website needs to be adjusted based on Iranian cultural and religious context to address core concerns of HPV-positive women discussed in this study.

It has been suggested that Iranian health insurance companies should be committed to including CC screening (Pap smear and HPV-testing) in their basic service packages [31]. It needs to be noted that some patients preferred not to use services covered by insurance and were concerned about the negative consequences of HPV-related insurance records. These finding demonstrated how evident and severe the stigma is in this setting.

Some of the findings are specific to religious, social, and cultural norms and cannot be generalized to other communities. According to McCaffery, HPV testing does represent a conflict for women from specific cultural and religious backgrounds [32]. It seemed Iranian women have solved that conflict by attributing the HPV-transmission to non-sexual routes. Factual information should be provided to avoid misconceptions. Providers must keep this in mind that in Iranian culture and society, premarital sex is often not common and accepted for women. Stigma, in some cases, affects receiving health care services. Healthcare professionals need to understand women and treat them respectfully and with no judgment.

In societies that HPV is common normalization can help de-stigmatization [33] but in our setting, it cannot be applied. Thus, stigma tend to experience more intensely.

The studies, though limited, verified low health literacy among Iranians related to HPV [34]. Promoting health literacy, especially women's education on sexual reproductive health, should be highlighted in the Iranian health education system. It can mitigate adverse effects of receiving HPV positive results on women's psychosocial well-being by de-stigmatising STDs. Our study demonstrated that women felt less stigmatised and receive more emotional support being in online groups with other HPV-positive patients. Given that, the Internet can provide a source of support for many individuals with HPV [35]. Forming interactive online HPV communities is recommended.

The most concern of participants who were mothers was related to contaminating their children. Since Sabeena publicized “oral HPV infection appears to be significant in the transfer of HPV among family members” [36], the worries over HPV contracting are not pointless. They should be educated in preventive behaviors and encouraged to vaccinate their teens.

Interviews showed women take boosting their immune system as a treatment. Also, despite FDA^6^ recommendation, most participants and even their partner had started vaccination against types they were not infected with.

Since no cure for HPV is presently available, the recommendations toward the treatment of HPV-positive patients are often conservative management with a close follow-up. Although WHO has mentioned that CC is mostly preventable [37], a limited number of our participants believed that they can prevent CC. Women reported lifestyle modifications to boost their immune system after their positive CC screening test. As mentioned in previous studies, cancer screening setting can work as a valuable opportunity to “Teachable moment,” (TM) for promoting healthy lifestyles [38, 39]. The bottom-line is we must ensure that women fully understand what it means to test hrHPV, which makes the goals of screening achievable for the benefit of both the patients and screening providers.

### Study limitations

Interviews were carried out over a fifteen-month period, due to researcher availability. However, we do not think this would have affected our findings as there was no major news or online media coverage of HPV during this time period, or change in health professional communication guidance.

### Clinical implications

Results indicate that healthcare providers need to know women's negative psychological responses to positive HPV result and address their concerns by providing more attention and support.

Some considerations are needed to how HPV-information is conveyed since misinformation received from invalid sources amplified women's fear of cancer. Attention should be paid to questions and concerns of women with mixed-status since they may experience more rejection and fear of spreading the virus. Asking about concurrent genital warts in hrHPV screening is suggested. The participants who were mothers feared of contaminating their children, so they should be educated in preventive behaviors and encouraged to vaccinate their teens. Being in online groups with other HPV-positive patients can lessen stigma and provide emotional support from women. Lifestyle modification to boost immunity to clear HPV indicated that the positive results of screening may have the potential to enhance women's physical and sexual health. Health authorities can provide multidisciplinary self-care guidance, including healthy eating, regular sleep, safe sex, and regular follow-up to promote women empowerment and shared decision making in the process of cervical cancer screening. More research is suggested to determine factors associated with adverse psychological responses to the positive result of HPV.

## Conclusion

This study provides an overview of negative psychological responses to hrHPV positivity. Women with HPV are in a difficult situation and need support and factual information. Providing deep understanding and wider resonance, these findings can help health authorities and policymakers of countries that are in the process of adopting HPV testing for cervical screening. In addition to cervical cancer, other HPV-associated cancers need to be taken into account when medical professionals are counseling HPV-positive women. Therefore, clinicians and other medical professionals involved in the HPV testing, need to be prepared to provide HPV-positive women with update comprehensive information, and emotional support. In conclusion, findings may help clinicians and educators to create an evidence-based educational programme or psychological-based intervention to improve women's physical, mental and sexual health.

## Supplementary Information

**Additional file 1:** Interview guide.

## Data Availability

The data that support the findings of this study are available from the corresponding author, [STM], upon reasonable request.

## Footnotes

1 Cervical Cancer Screening Program.

2 Cervical Intraepithelial Neoplasia.

3 A female pilgrim to Mecca.

4 Centers for Disease Control.

5 World Health Organization.

6 Food and Drug Administration.
