# Supplementary material for: Iranian women’s psychological responses to positive HPV test result: a qualitative study
Source: BMC Womens Health. 2021 Mar 26;21:128. doi: 10.1186/s12905-021-01272-x (PMC7995699; doi:10.1186/s12905-021-01272-x)
Supplement: Supplementary file 1 — Additional file 1: Interview guide. [file 12905_2021_1272_MOESM1_ESM.docx]

Iranian Women's Psychological Responses to positive HPV test result: A Qualitative Study

Running Title: Psychological responses to positive HPV test result

Kowsar Qaderi^, Seyedeh Tahereh Mirmolaei^, Mehrnaz Geranmayeh^, Shahrzad Sheikh Hasani**, Farnaz Farnam^

**Correspondence** should be sent to: [mirmolaei@tums.ac.ir](mailto:mirmolaei@tums.ac.ir)

Seyedeh Tahereh Mirmolaei, Nursing and Midwifery School, Eastern Nosrat st. Tohid sq. Tehran I.IRAN 141973317 +989122245100,

**Appendix 1**

| Interview guide: |
| --- |
| *“What have you been told at the time of receiving positive HPV test results or what do you know about HPV?”*  *“How did you feel and how was your reaction at the time of receiving result?”*  *“Please tell me about your experiences, thoughts, and initial feelings?” “Are you experiencing any concerns?”*  *“How is your personal and emotional life affected by the HPV test results?”*  *“How about disclosing HPV to partner/others and their reactions?”* |
